# Effectiveness of 13-pneumococcal conjugate vaccine (PCV13) against invasive pneumococcal disease in children in the Dominican Republic

**DOI:** 10.1186/s12879-018-3047-3

**Published:** 2018-04-02

**Authors:** Sara Tomczyk, Fernanda C. Lessa, Jacqueline Sánchez, Chabela Peña, Josefina Fernández, M. Gloria Carvalho, Fabiana Pimenta, Doraliza Cedano, Cynthia G. Whitney, Jennifer R. Verani, Hilma Coradin, Zacarías Garib, Lucia Helena De Oliveira, Jesús Feris-Iglesias

**Affiliations:** 1 Division of Bacterial Diseases, Centers for Disease Control and Prevention, Atlanta, GA USA; 2 Epidemic Intelligence Service, Centers for Disease Control and Prevention, Atlanta, GA USA; 3 Department of Infectious Diseases, Dr. Robert Reid Cabral Children’s Hospital, Santo Domingo, Dominican Republic; 4 Programa Ampliado de Inmunizaciones, Ministerio de la Salud Pública, Santo Domingo, Dominican Republic; 5 Pan American Health Organization, Washington, DC USA

**Keywords:** Pneumococcal conjugate vaccine, Effectiveness, Case-control study, Invasive pneumococcal disease

## Abstract

**Background:**

Limited data are available on the effectiveness of 13-valent pneumococcal conjugate vaccine (PCV13) in resource-poor settings and PCV naïve populations. The Dominican Republic introduced PCV13 in September 2013 using a 2 + 1 schedule (2, 4, and 12 months) without a catch-up campaign. We evaluated PCV13 effectiveness against vaccine-type (VT) invasive pneumococcal disease (IPD) among children in the Dominican Republic.

**Methods:**

We conducted a matched case-control study. A case-patient was defined as VT-IPD identified by culture or polymerase chain reaction (PCR) from a normally sterile-site in a hospitalized child who was age-eligible to have received ≥1 PCV13 dose. Four age- and neighborhood-matched controls were enrolled for each case-patient. We collected demographic, vaccination history, and risk factor data. Conditional logistic regression was performed. Vaccine effectiveness was calculated as (1- adjusted matched odds ratio for vaccination) X 100%.

**Results:**

We enrolled 39 case-patients and 149 matched-controls. Most case-patients had pneumonia with pleural effusion (64%), followed by meningitis (28%) and septicemia (13%). The most common pneumococcal serotypes identified included 14 (18%), 3 (13%), 19A (10%), and 1 (8%). Fewer case-patients had ≥1 PCV13 dose as compared to controls (61.5% vs. 80.0%; *p* = 0.006). Adjusting for malnutrition and socioeconomic status, VE of ≥1 PCV13 dose compared to no doses was 67.2% (95% CI: 2.3% to 90.0%). Only 44% of controls were up-to-date for PCV13, suggesting low vaccine coverage in the population.

**Conclusions:**

We found that PCV13 provided individual protection against VT-IPD in this resource-poor setting with a PCV-naïve population, despite low PCV13 coverage. Expanding vaccination coverage might increase PCV13 impact.

## Background

*Streptococcus pneumoniae* is a leading cause of vaccine-preventable death in children less than five years of age [1]. *S. pneumoniae* can cause pneumonia, otitis media, sinusitis and invasive pneumococcal disease (IPD) like bacteremia and meningitis. The pneumococcal conjugate vaccine (PCV) was identified by the World Health Organization as an important public health intervention to prevent deaths due to pneumococcal disease in developing countries [2]. Approximately 70 to 80% of IPD cases among children less than five years old in Latin America are caused by pneumococcal serotypes included in the ten-valent (PCV10) or thirteen-valent (PCV13) vaccines [3].

The first generation PCVs (PCV7 and PCV9) were shown to be effective in clinical trials and in practice [4–8], but limited data are available on the second generation PCVs (PCV10 and PCV13), in particular from low- and middle-income countries where three-dose schedules for PCVs have been implemented. Evaluation of second generation PCVs conducted in South Africa (PCV13) and Brazil (PCV10) have demonstrated the effectiveness of PCV against IPD in these settings. However, in South Africa the population was not PCV naïve because PCV13 replaced PCV7 in 2011 and the country was also the site of a PCV clinical trial, while in Brazil, a catch-up campaign and four doses of PCV were used, rather than the more-commonly used 3-dose schedule [9–11]. Thus, questions still remain about the performance of PCV in a “typical” resource-poor setting, including the effectiveness of PCV13 in a population with no prior PCV use (i.e. PCV naïve populations) using a three dose (i.e., 2 + 1) schedule.

In August 2013, PCV13 was introduced in the Dominican Republic using a 2-dose primary series (at 2 and 4 months) plus a booster dose at 12 months. No catch-up campaign was conducted, and neither PCV7 nor PCV10 had been used previously. Prior to PCV13 introduction, approximately 90% of cases of pneumococcal pneumonia with effusion in children admitted to the infectious diseases service were caused by the serotypes included in PCV13 [12]. *S. pneumoniae* is the most common cause of pneumonia with pleural effusion among children admitted to the infectious disease service [12]. This study aimed to determine the effectiveness of PCV13 against vaccine-type IPD among eligible children in this setting.

## Methods

### Study population and design

The Dominican Republic is a middle-income country in the Caribbean with a population of 10.3 million, and a birth rate of 21 births per 1000 people (2013) [13]. The study was conducted at the national reference pediatric hospital in the Dominican Republic, a pediatric referral tertiary care hospital located in the capital city Santo Domingo. IPD surveillance began at the hospital in 2000 with a focus on pediatric meningitis and pneumonia with effusion.

The study used a matched case-control design. A case-patient was defined as a vaccine type (VT)-*S. pneumoniae* (i.e. serotypes included in PCV13) identified from a normally sterile-site specimen (i.e. cerebrospinal, blood, joint, or pleural fluid) in a hospitalized child who was age-eligible to have received at least one dose of PCV13 (i.e. at least 2 months old on admission and born on or after July 1, 2013), and not previously enrolled in the study. Study staff reviewed admission logs and microbiology records daily to identify potential case-patients.

For each case-patient, we aimed to enroll at least four controls matched on age and neighborhood of residence. Children were eligible to be enrolled as controls if they were a resident of the case-patients’s neighborhood for at least one month prior to the matched case-patient’s hospital admission date, age-eligible to have received at least one dose of PCV13 by the time of hospital admission for the case-patient, not hospitalized during the prior month for pneumonia, meningitis or sepsis, and not previously enrolled in the study. Using the case-patient’s residence as a starting point, controls were sought house-to-house using a random walk approach commonly used for vaccine coverage surveys [14]. Up to three attempts were made to reach households targeted for enrollment. A registry was kept of all households approached.

Prior to enrollment, written informed consent was obtained from the parent or guardian of all potential study participants. Socio-demographic, risk factor, and clinical data were collected from study participants by interviews with the parents or guardians using a standardized questionnaire. Medical records were also reviewed to abstract case-patient data, including anthropometric data. For control anthropometric data, study staff measured the height of the children and recorded the weight based on records supplied by the parents or guardians. Written evidence of immunization history was actively sought and examined. The primary source of vaccine history was the child immunization card; if that was not available, parents’ verbal vaccine information was confirmed through vaccination records at immunization clinics. Those with no written documentation of any vaccine receipt (e.g. no immunization card available and vaccine history could not be confirmed in vaccination records at immunization clinics) were excluded from the analysis unless the parent or guardian reported that their child had not received any vaccinations beyond doses given at birth (such children were included and considered to have received zero doses of PCV13).

Children were included as controls if the age-matching criteria were confirmed and their birthdate was within 51.4 days or 81.8 days of the matched case-patients’ birthdate for case-patients aged less than and greater than or equal to six months, respectively. Criteria were originally set at 1 month (30.4 days) and 2 months (60.8 days), respectively, but were revised to add 21 days to for each group to address challenges faced by field research staff with finding controls meeting these criteria, especially in neighborhoods with low population density. We defined a reference date for the controls that matched the time of hospital admission for the corresponding case-patient.

### Laboratory methods

Specimens were collected from patients for diagnosis as per routine medical care. Invasive specimens were plated on 5% sheep blood agar and incubated overnight in CO2 at 37 degrees Celsius. Suspect colonies were identified as *S. pneumoniae* by susceptibility to optochin and bile solubility tests. Suspect alpha-hemolytic colonies were identified as *S. pneumoniae* by susceptibility to optochin and/or bile solubility tests. Cerebrospinal fluid (CSF), pleural, and joint fluids were tested for presence of pneumococcal antigen by immune-chromatographic test BinaxNow (i.e. Binax) and for presence of the pneumococcal *lytA* gene by quantitative PCR [15]. Pneumococcal isolates were serotyped by Quellung, while *lytA* positive specimens that were culture-negative were serotyped by quantitative multiplex PCR targeting 37 serotypes (including VT serotypes) [16, 17]. To obtain deoxyribonucleic acid (DNA) extracts for polymerase chain reaction (PCR) reactions, 200–400 μL of the clinical specimens were transferred into 1.5 ml cryotubes containing 300 μL of buffer#4 (Roche isolation kit III) and transferred to MagNA Pure Compact instrument using the external lysis protocol according to manufacture instructions. When samples were viscous or containing clots or fragments, 200–300 μl of ATL Buffer (Qiagem) and 30 min incubation at 56 °C treatment was added to dissolve the fragments. DNA extracts were eluted to 100 μL and stored at -20 °C until PCR testing was performed using Quanta Biosciences PerfeCTa® qPCR ToughMix®, Low ROX™, for the *lyt*A assay and PerfeCTa Multiplex qPCR ToughMix®, Low ROX™, for the multiplex serotyping assays [15].

### Data analysis

Vaccine doses were considered to be valid only if given on or after 6 weeks of age, at least 21 days after the previous PCV dose, and at least 14 days before hospital admission for case-patients (or the reference date for controls). An up-to-date PCV13 variable was generated if the number of valid doses was at least one dose for children aged ≤4 months and 13 days, at least two doses for children aged between 4 months plus 14 days and ≤ 12 months plus 13 days, and at least three doses for children ≥12 months plus 14 days at the time of hospital admission or reference day. Serotyping by PCR does not distinguish between the serotypes within the following serogroups: 6A/6B, 7F/7A, and 18C/18F/18B/18A; thus, we classified these according to their serogroup in the analysis. Low weight-for-age Z score was defined as <− 2 using World Health Organization (WHO) growth curves [18]. We used multivariable conditional logistic regression to calculate the matched odds ratio of PCV13 vaccination. Matched pairs with at least one discordant set (i.e. different vaccination status between case-patients and controls) contributed to the models. Vaccine effectiveness (VE) was calculated as (1- adjusted matched odds ratio) X 100%. We assessed confounding by adding variables to the model one-by-one. Any variables which changed the VE estimate by more than 10% were included. Serotype-specific VE models were explored where there was sufficient power. A sensitivity analysis was also performed by excluding case-patients that were positive by PCR for serogroups that included a non-pneumococcal vaccine-type serotype. Data were analyzed using SAS software (version 9.3; SAS Institute).

The protocol was approved by the ethics committees of Hospital Infantil Robert Reid Cabral and the Pan American Health Organization (PAHO). The Centers for Disease Control and Prevention (CDC) determined the protocol to be public health non-research.

## Results

Fifty patients eligible for case-patient enrollment were identified with IPD onset from December 2013 through January 2016. Study implementation began in May 2014, and eligible case-patients with disease onset before May 2014 (*n* = 5) were retrospectively enrolled. Of the total 50 eligible IPD case-patients, 11 (22.0%) were not enrolled because they were discharged from the hospital before consent was obtained and no contact information was available in the medical record to enable follow-up (*n* = 10), or no documentation of vaccination history could be obtained despite parents reporting vaccine receipt after birth (*n* = 1). Therefore, a total of 39 case-patients, median age of 10.1 months (interquartile range: 5.3–17.4 months old), were included in the study. A total of 164 controls were initially recruited for the 39 enrolled case-patients (31 case-patients with 4 controls and 8 case-patients with 5). However, 15 were excluded because they were not age-eligible (*n* = 5), did not meet age-matching criteria (*n* = 8), or had no documentation of vaccine history (*n* = 2). Thus, a total of 149 controls, median age of 9.9 months (interquartile range: 4.8–15.3 months) were included in the study; 6 case-patients (15%) were matched to < 4 controls.

Among enrolled case-patients and controls, approximately one-third were younger than 6 months, one-half were female, and one-fifth had a history of comorbidities (Table 1). Families of case-patients were significantly less likely to own a home (18% vs. 50%; *p* = 0.0002) and more likely to live in a home built of wood as compared to control’s families (31% vs. 13%; *p* = 0.01). More case-patients lived in a home with ≥1 child < 5 years of age (44% vs. 28%; *p* = 0.09), although fewer case-patients lived in homes with > 2 persons/room (i.e. crowding index) (36% vs. 59%; *p* = 0.01) as compared to controls. Case-patients were significantly more likely to be malnourished (i.e. low weight-for-age Z score) as compared to controls (28% vs. 9%; *p* = 0.003).

**Table 1:** Sociodemographic and clinical characteristics of invasive pneumococcal disease (IPD) case-patients and community controls

Factor	Case-patient (%) *N* = 39	Control (%) *N* = 149	Matched *p*-value
Age < 6 months	12 (31)	47 (32)	1.00
Female sex	17 (44)	74 (50)	0.54
Born in the Dominican Republic	38 (97)	145 (97)	1.00
Lives in urban area	26 (67)	102 (68)	1.00
Family owns a home	7 (18)	75 (50)	0.0002
Living conditions			
Home with dirt floor^a^	2 (5)	4 (3)	0.73
Home built of wood^a^	12 (31)	19 (13)	0.01
Has electricity	1 (3)	3 (2)	1.00
Has indoor bathroom	23 (59)	107 (72)	0.14
Means of cooking			
Coal	1 (3)	21 (14)	0.06
Gas	36 (92)	139 (93)	1.00
Firewood	3 (8)	16 (11)	0.69
Cooking done in room where others congregate or sleep	12 (31)	23 (15)	0.08
Lives with ≥1 child < 5 years	17 (44)	42 (28)	0.09
Crowding index (> 2 persons/room)	14 (36)	88 (59)	0.01
Breastfed in the last month	18 (46)	50 (34)	0.12
History of ≥1 household member smoking inside in the last month	4 (10)	21 (14)	0.76
Mother completed primary school	26 (67)	115 (77)	0.22
Low birthweight (< 2500 g)	3 (8)	14 (9)	1.00
Born premature (< 36 weeks)	1 (3)	8 (5)	0.88
Low weight-for-age Z score (<−2 SD)	11 (28)	13 (9)	0.003
History of comorbidities	7 (18)	33 (22)	0.71
History of another infection in last 12 months	15 (38)	47 (32)	0.20
History of medical consultation for respiratory infection in last 12 months	32 (82)	133 (89)	0.46
History of cold or cough in the last month	21 (54)	124 (83)	0.001
Up-to-date with PCV^b^	11 (28)	66 (44)	0.02
Number of valid PCV doses			
0	15 (38)	30 (20)	–
1	6 (15)	44 (30)	0.01
2	18 (46)	59 (40)	0.25
3	0 (0)	16 (11)	0.002

^a^Homes with dirt floors and built of wood are considered lower quality materials (i.e. low socioeconomic proxy) compared to reference categories of higher quality material, i.e. cement, brick

^b^One valid PCV dose for children aged ≤4 months and 13 days, at least two valid doses for children aged between 4 months plus 14 days and ≤ 12 months plus 13 days, and at least three valid doses for children ≥12 months plus 14 days at the time of hospital admission or reference day

The most common clinical presentations among case-patients included pneumonia with pleural effusion (64%), meningitis (28%), and septicemia (13%). Binax tests were positive in 8 (80%) of the 10 CSF samples tested and in 22 (92%) of the 24 pleural fluid samples tested. All four case-patient specimens that were Binax negative were also culture negative, but were PCR-positive for pneumococcal *lytA* gene. The most prevalent pneumococcal serotypes identified in case-patient clinical specimens were 6A/6B (31%), 14 (18%), 3 (13%), and 19A (10%) (Table 2). Among case-patients who received two valid doses of PCV13, serotype 3 (*n* = 4; 22%), 14 (*n* = 3; 17%), and 19A (*n* = 3; 17%) were most common. Approximately one-half of case-patients were referred from another hospital. Two case-patients died (Table 2).

**Table 2:** Clinical characteristics of IPD case-patients (*N* = 39)

Characteristics	Number (%)
Syndrome	
Pneumonia with pleural effusion	25 (64)
Meningitis	11 (28)
Septicemia	5 (13)
Bacteremic pneumonia	2 (5)
Culture positive for pneumococcus	
Blood	8 (21)
Cerebrospinal Fluid (CSF)	8 (21)
Binax positive	
CSF, positive/tested	8/10
Pleural fluid, positive/tested	22/24
Pneumococcal serotype identified	
6A/6B	12 (31)
14	7 (18)
3	5 (13)
19A	4 (10)
1	3 (8)
19F	2 (5)
Serogroup 18	2 (5)
4	2 (5)
7F/7A	1 (3)
Referred from another hospital	21 (54)
Died	2 (5)

**Fig. 1:**
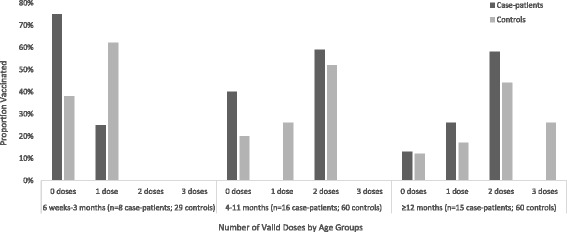
Number of valid PCV doses by age group and case-patient status

Fewer case-patients had received ≥1 PCV13 dose as compared to controls (*n* = 24, 61.5% vs. *n* = 119, 80.0%; *p* = 0.006) and fewer case-patients were up-to-date with PCV13 (*n* = 11, 28% vs. *n* = 66, 44%; *p* = 0.02) (Table 1). Across age groups 0–3 months, 4–11 months, and ≥ 12 months, more case-patients were reported to have received no doses of PCV, and none of the case-patients received three doses of PCV (Fig. 1). Adjusting for low weight-for-age Z score (i.e. malnutrition) and home built of wood (i.e. socioeconomic proxy), VE of ≥1 valid PCV dose compared to no doses was 67.2% (95% CI: 2.3% to 90.0%); 23 out of 39 case-control sets were discordant and contributed to this model. This adjusted estimate did not change after excluding case-control sets where PCR could not distinguish between vaccine and non-vaccine types in certain serogroups of case-patients. The adjusted estimate increased slightly after restricting the analysis to children born in the Dominican Republic (69.9%; 95% CI: 11.9% to 90.6%). The only serotype-specific VE model with sufficient power for analysis assessed the risk of serotype 6A/6B disease for children with ≥1 compared to no valid PCV doses, and showed an adjusted VE of 93.3%; 95% CI: 13.0% to 99.9%) (Table 3).

**Table 3:** Effectiveness of pneumococcal conjugate vaccine schedule against VT-IPD

Outcome	Discordant pairs^a^	Unadjusted (95% CI)	Adjusted (95% CI)^b^
≥1 valid PCV dose compared to no doses			
Overall	23/39	68.6% (14.5% to 89.2%)	67.2% (2.3% to 90.0%)
Born in Dominican Republic	23/39	70.6% (20.4% to 89.8%)	69.9% (11.9% to 90.6%)
Serotype 6A/6B ^c^	7/12	93.3% (41.6% to 99.9%)	93.3% (13.0% to 99.9%)
Up-to-date with PCV compared to no doses ^d^	17/39	71.7% (− 7.2% to 94.0%)	68.6% (− 29.6% to 93.9%)

^a^Matched pairs with at least one discordant set (i.e. different vaccination status between case-patient and control). In conditional logistic regression, only discordant pairs contribute to the model

^b^Adjusted for low weight-for-age Z score (i.e. malnutrition) and home built of wood (i.e. socioeconomic proxy)

^c^Only able to evaluate serotype 6A/6B because of lack of power to evaluate vaccine effectiveness for other individual serotypes

^d^Up-to-date PCV13 was at least one dose for children aged ≤4 months and 13 days, at least two doses for children aged between 4 months plus 14 days and ≤ 12 months, and at least three doses for children > 12 months plus 14 days old at the time of hospital admission or reference day

## Discussion

PCV13 introduction into the routine national immunization program in the Dominican Republic was effective in the prevention of VT-IPD. Although PCVs, especially PCV7, are known to be efficacious based on clinical trials and observational studies in developed countries [4–7, 19, 20], our study addresses the performance of PCV13 in a middle-income setting in Latin America and in a PCV-naïve population where data are still limited. The most prevalent serotypes causing IPD in the Dominican Republic during our post-PCV introduction evaluation were 6A/6B, which, prior to the introduction of pneumococcal vaccine, were second only to serotype 14 as a cause of IPD in children < 5 years of age Latin America [3]. We were able to show high PCV13 effectiveness (VE: 93.3%) against IPD caused by serotypes 6A/6B.

The adjusted VE point estimate of 67.2% for at least one dose of PCV13 was lower than the estimate of 81.9% found in the study by Domingues et al. in Brazil after the introduction of PCV10 using a four dose schedule [10], although confidence intervals from the two studies widely overlap. Another study by Cohen et al. in South Africa from 2010 to 2015 did not find a significant effect of one dose of PCV7 or PCV13 (adjusted VE of 1%; 95% CI: -141% to 59%) given at approximately 6 weeks among children without human immunodeficiency virus (HIV), but did find a significant effect of at least two doses of PCV13 (85%, 95%CI: 37%–96%) against VT-IPD [11]. The low number of control children who were up to date for PCV13 doses limited our ability to assess vaccine effectiveness specifically for complete schedules or comparing two versus three doses.

We found that case-patients were significantly more likely than controls to be malnourished (*p* = 0.003), and we therefore controlled for malnutrition in our adjusted VE models. PCVs have been found to have decreased effectiveness among malnourished children in other low- to middle-income country settings, although these data are also limited and heterogeneous [9, 11, 21] and the explanation for this effect is unknown.

While we found PCV13 to be effective, its impact, both directly among young children eligible for vaccination and indirectly among older children and adults through indirect (herd) effects, was likely limited by low vaccine coverage. Only 44% of the control-patients in our study were up-to-date with PCV based on their age, and data from the Dominican Republic Ministry of Health indicated low PCV13 coverage, especially of the third dose, due to challenges in the supply chain. Coverage of the 3rd dose of PCV13 was 27 and 22% in 2014 and 2015, respectively, while the coverage for the 3rd dose of the Diphtheria-Tetanus-Pertussis (DTP) vaccine was 91 and 85% for the same periods [22]. The Dominican Republic is not a Global Alliance for Vaccines and Immunization (GAVI)-eligible country [23], so it does not have external support from donors for vaccine purchase. The cost of each dose of PCV13 via the PAHO Revolving Fund, a fund that purchases vaccines for 41 countries and territories as a group to improve purchasing power and drive vaccine costs down, is $14.50 compared to $0.20 for DPT [24]. PCV is the most expensive vaccine in the routine infant immunization schedule, and even with a reduced cost per PCV dose, some middle-income countries are struggling to maintain high coverage levels [22, 25]. The low PCV vaccination coverage in the Dominican Republic is of concern and strategies to increase PCV coverage should be considered. More PCV manufacturers in the market could help drive down prices, which is particularly important for non-GAVI eligible countries or recently graduated GAVI countries [26].

The study environment offered several strengths. The Dominican Republic had a PCV-naïve population prior to the introduction of PCV13, in contrast to other settings where PCV7 was introduced prior to PCV13, making evaluation of PCV13 more difficult. The study was also conducted at the largest tertiary pediatric hospital in the country, which enabled more complete case-patient ascertainment. However, there were several study limitations to consider. Although matching case-patients and controls by neighborhood potentially controls for confounders such as socioeconomic status, ethnic origin, and geographic location, it may have led to over-matching on the exposure of interest (i.e. PCV13 receipt) due to local challenges with the vaccine supply chains and distribution. Of the 39 matched case-control sets enrolled, only 23 (59%) were discordant (i.e. vaccine exposure different between case-patient and controls) and contributed to the final effectiveness model. Lastly, delays in interviewing controls may have led to recall bias for some of the information collected, although attempts were made to identify controls within 3 months of case-patient admission. Use of written vaccination records, however, should have limited bias for history of PCV13 receipt.

## Conclusions

Routine use of PCV13 as part of the infant immunization schedule in the Dominican Republic was effective in preventing invasive disease caused by vaccine-types, demonstrating the benefit of PCV introduction in similar settings. Given the burden of pneumococcal disease in such countries, our study highlights the use of PCV as an important public health intervention. Sustained PCV use and high population coverage will be needed to ensure optimal effectiveness.

## Abbreviations

CDC: Centers for Disease Control and Prevention
CSF: Cerebrospinal fluid
DNA: Deoxyribonucleic acid
DTP: Diphtheria-tetanus-pertussis
GAVI: Global Alliance for Vaccines and Immunization
HIV: Human immunodeficiency virus
IPD: Invasive pneumococcal disease
PAHO: Pan American Health Organization
PCR: Polymerase chain reaction
PCV: Pneumococcal conjugate vaccine
PCV10: Ten-valent pneumococcal conjugate vaccine
PCV13: Thirteen-valent pneumococcal conjugate vaccine
PCV7: Pneumococcal conjugate vaccine
PCV9: Nine-valent pneumococcal conjugate vaccine
VE: Vaccine effectiveness
VT: Vaccine type
WHO: World Health Organization
